# Identification of a novel microRNA recurrence-related signature and risk stratification system in breast cancer

**DOI:** 10.18632/aging.102268

**Published:** 2019-09-23

**Authors:** Jianguo Lai, Bo Chen, Guochun Zhang, Yulei Wang, Hsiaopei Mok, Lingzhu Wen, Zihao Pan, Fengxi Su, Ning Liao

**Affiliations:** 1 Department of Breast Cancer, Cancer Center, Guangdong Provincial People’s Hospital and Guangdong Academy of Medical Sciences, Guangzhou, China; 2 Guangdong Provincial Key Laboratory of Malignant Tumor Epigenetics and Gene Regulation, Sun Yat-Sen Memorial Hospital, Sun Yat-Sen University, Guangzhou, China; 3 Breast Tumor Center, Sun Yat-Sen Memorial Hospital, Sun Yat-Sen University, Guangzhou, China; 4 Department of Thoracic Surgery, Sun Yat-Sen Memorial Hospital, Sun Yat-Sen University, Guangzhou, China

**Keywords:** breast cancer, microRNA, model, survival, recurrence

## Abstract

Increasing evidence has revealed that microRNAs (miRNAs) play vital roles in breast cancer (BC) prognosis. Thus, we aimed to identify recurrence-related miRNAs and establish accurate risk stratification system in BC patients. A total of 381 differentially expressed miRNAs were confirmed by analyzing 1044 BC tissues and 102 adjacent normal samples from The Cancer Genome Atlas (TCGA). Then, based on the association between each miRNAs and disease-free survival (DFS), we identified miRNA recurrence-related signature to construct a novel prognostic nomogram using Cox regression model. Target genes of the four miRNAs were analyzed via Gene Ontology and KEGG pathway analyses. Time-dependent receiver operating characteristic analysis indicated that a combination of the miRNA signature and tumor-node-metastasis (TNM) stage had better predictive performance than that of TNM stage (0.710 vs 0.616, *P*<0.0001). Furthermore, risk stratification analysis suggested that the miRNA-based model could significantly classify patients into the high- and low-risk groups in the two cohorts (all *P*<0.0001), and was independent of other clinical features. Functional enrichment analysis demonstrated that the 46 target genes mainly enrichment in important cell biological processes, protein binding and cancer-related pathways. The miRNA-based prognostic model may facilitate individualized treatment decisions for BC patients.

## INTRODUCTION

Breast cancer (BC) is most commonly malignancy and lead to main cancer death among females worldwide [1–4]. Although the improvement of BC prognosis has been made, most of BC-related deaths are caused by tumor relapse or progression [5]. BC has been recognized as a heterogeneous disease that displays distinct differences in respect of biological behavior, gene expression profiles and survival outcome, even in the same tumor-node-metastasis (TNM) stage [6]. Traditionally, the TNM classification is important tool for prognostic assessment and treatment decisions. However, the TNM system has some limitations. First, patients with equivalent anatomical spread yet variable survival outcome are assigned into the same stage, ignoring the heterogeneity of BC [7]. Second, the TNM system is unable to integrate other important prognostic risk factors, such as lymphovascular invasion (LVI), histological grade, Ki67, and molecular markers. Third, the TNM system could not achieve individualized risk prediction of survival for each patient. Hence, there is an urgent clinical need to establish a practical tool incorporating molecular markers and other prognostic factors for accurately predicting survival in BC patients.

MicroRNAs (miRNAs) are small, noncoding RNAs that regulate multiple cellular processes, such as cell differentiation, apoptosis, and cell-cycle progression [8, 9]. A growing number of researches have shown that the differentially expressed miRNAs (DEMs) might serve as prognostic molecular biomarkers for various tumors [10–20]. Traditional clinicopathological risk factors were unable to clearly distinguish between BC patients who have a low or high risk of relapse. Thus, a comprehensive approach of integrating DEMs and other prognostic factors to achieve reliable risk stratification is highly necessary. Nomogram, which is a visual statistical model, can provide a numerical probability of a clinical event for each patient [21]. Furthermore, nomogram enables to make individualized estimates of survival to aid clinical decision making.

Consequently, we aimed to develop and validate a novel multi-miRNA-based model incorporating recurrence-related miRNAs and other risk factors for evaluating disease-free survival (DFS) and make effective risk stratification in BC patients.

## RESULTS

### Patient characteristics

In this study, a total of 897 patients with invasive BC from The Cancer Genome Atlas (TCGA) database were selected. Table 1 showed baseline characteristics of the derivation, and the internal validation sets. No significant difference of baseline characteristics were observed between the two data sets (all *P* > 0.05). The median age of the patients were 58 year (interquartile range [IQR]: 48–66) and 56 year (IQR: 47–66) in the two independent cohorts. The 5-year DFS rates of the patients were 0.823 (95% CI: 0.780–0.858) and 0.864 (95% CI: 0.809–0.905) in the derivation and the internal validation cohort, respectively.

**Table 1:** Baseline characteristics of study patients.

**Variables**	**Derivation cohort**	**Validation cohort**	***P* value**
	**No. (%)**	**No. (%)**	
**No. of patients**	897	449	
**Age (years)**	58 (48, 66)	56 (47, 66)	0.572
**T stage**			0.730
T1	242 (27.0)	122 (27.2)	
T2	524 (58.4)	266 (59.2)	
T3	111 (12.4)	48 (10.7)	
T4	20 (2.2)	13 (2.9)	
**N stage**			0.980
N0	426 (47.5)	214 (47.6)	
N1	305 (34.0)	149 (33.2)	
N2	94 (10.5)	47 (10.5)	
N3	66 (7.3)	35 (7.8)	
Nx	6 (0.7)	4 (0.9)	
**TNM stage**			0.806
I	159 (17.7)	86 (19.1)	
II	523 (58.3)	255 (56.8)	
III	205 (22.9)	105 (23.4)	
IV	10 (1.1)	3 (0.7)	
**ER status**			0.998
Negative	187 (20.8)	93 (20.7)	
Positive	676 (75.4)	339 (75.5)	
Unknown	34 (3.8)	17 (3.8)	
**PR status**			0.801
Negative	241 (26.9)	117 (26.1)	
Positive	551 (61.4)	274 (61.0)	
Unknown	105 (11.7)	58 (12.9)	
**Her2 status**			
Negative	634 (70.7)	316 (70.4)	0.945
Positive	136 (15.1)	71 (15.8)	
Unknown	127 (14.2)	62 (13.8)	

TNM, tumor-node-metastasis; ER, estrogen receptor; PR, progesterone receptor; Her2, human epithelial growth factor receptor 2.

### Construction of miRNA-based risk score and prognostic model

**Figure 1:**
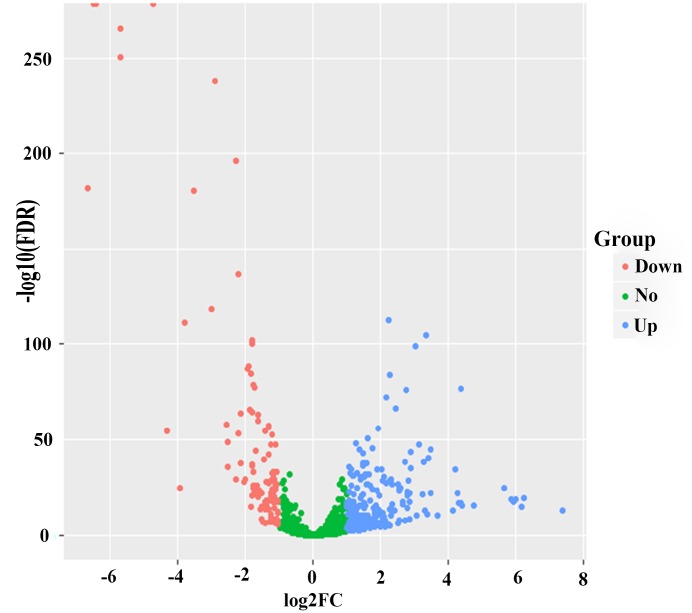
**Volcano plot of 273 up-regulated and 108 down-regulated.** Blue color represents up-regulated expression, and red color reveals down-regulated expression.

**Figure 2:**
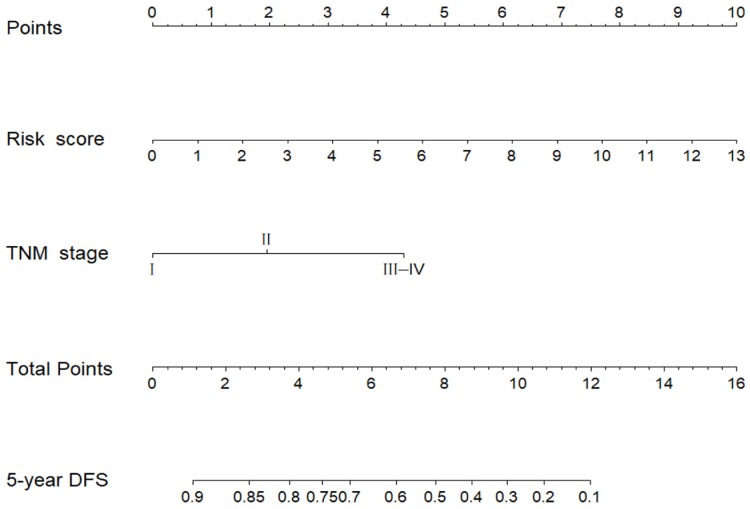
**miRNA-based prognostic model to predict 5-year disease-free survival in breast cancer patients.**

After edgeR filtering (false discovery rate [FDR] < 0.05 and log2 fold change [log2FC] ≥ 1) between 1044 BC samples and 102 adjacent normal tissues, we screened out 381 DEMs from 1601 miRNAs expression profiles. Of these DEMs, 273 miRNAs were upregulated, and 108 miRNAs were downregulated. And the 1601 miRNAs were visualized via volcano plot in Figure 1. Firstly, we found 13 DEMs (hsa-miR-488, hsa-miR-6125, hsa-miR-3651, hsa-miR-5691, hsa-miR-1276, hsa-miR-5008, hsa-miR-3178, hsa-miR-4522, hsa-miR-3145, hsa-miR-597, hsa-miR-1293, hsa-miR-219a-2, hsa-miR-4533) in univariate Cox proportional hazards regression (CPHR) analysis (*P* < 0.05). Finally, in light of multivariate CPHR analysis, four independent recurrence-related miRNAs (three risky miRNAs: hsa-miR-1293, hsa-miR-3145, hsa-miR-3178; one protective miRNA: hsa-miR-4522) were identified to construct a risk score in the derivation cohort (Table 2). Risk score = 0.495 × expression_hsa-miR-3145_ + 0.245 × expression_hsa-miR-3178_ + 0.100 × expression_hsa-miR-1293_ – 0.409 × expression_hsa-miR-4522_. The results of the univariate and multivariate CPHR analyses in the derivation cohort were listed in Table 3. On the basis of multivariate CPHR analysis, we confirmed two independent risk factors for DFS (*P* < 0.05), including TNM stage, and miRNA recurrence-related signature. It should be pointed out that T stage and N stage were associated with TNM stage, known as multicollinearity, could affect the beta coefficients on multivariate CPHR analysis, giving rise to spurious associations and unreliable results [21]. Therefore, T stage and N stage were not entered into multivariate CPHR analysis. To help clinicians with a quantitative tool for individualized risk prediction of DFS, we developed a novel prognostic nomogram that incorporated the miRNA recurrence-related signature and TNM stage to predict 5-year DFS in BC patients (Figure 2).

**Table 2:** miRNA recurrence-related signature in the derivation cohort.

**Name**	**Coefficient**	**Type**	**HR**	**95%CI**	***P* value**
**hsa-miR-1293**	0.100	Risky	1.105	1.041–1.172	0.001
**hsa-miR-3145**	0.495	Risky	1.640	1.250–2.151	<0.001
**hsa-miR-3178**	0.245	Risky	1.277	1.114–1.465	<0.001
**hsa-miR-4522**	−0.409	Protective	0.664	0.481–0.918	0.013

CI, confidence interval; HR, hazard ratio.

**Table 3:** Univariate and multivariate analyses in the derivation cohort.

**Variables**	**Univariate analysis**			**Multivariate analysis**	
	**Hazard ratios (95%CI)**	***P* value**		**Hazard ratios (95%CI)**	***P* value**
**Age**	1.008 (0.991–1.024)	0.362			
**T stage**					
T1	Referent				
T2	1.738 (0.903–3.346)	0.098			
T3/T4	3.665 (1.875–7.164)	**<0.001**			
**N stage**					
N0	Referent				
N1	1.596 (0.995–2.560)	0.053			
N2	2.125 (1.117–4.044)	**0.022**			
N3/Nx	5.454 (3.010–9.884)	**<0.001**			
**TNM stage**					
I	Referent			Referent	
II	1.738 (0.903–3.346)	0.098		1.743 (0.904–3.358)	0.097
III/IV	3.665 (1.875–7.164)	**<0.001**		3.477 (1.763–6.856)	**<0.001**
**ER status**					
Negative	Referent			Referent	
Positive	0.622 (0.404–0.960)	**0.032**		0.833 (0.462–1.501)	0.542
Unknown	1.030 (0.361–2.938)	0.956		0.923 (0.272–3.126)	0.897
**PR status**					
Negative	Referent			Referent	
Positive	0.598 (0.387–0.921)	**0.020**		0.706 (0.394–1.267)	0.243
Unknown	1.035 (0.555–1.929)	0.913		1.057 (0.508–2.200)	0.882
**Her2 status**					
Negative	Referent				
Positive	0.768 (0.394–1.496)	0.437			
Unknown	1.590 (0.973–2.600)	0.064			
**miRNA signature**	1.300 (1.181–1.431)	**<0.001**		1.207 (1.091–1.336)	**<0.001**

Bold values indicate statistical significance (*P*<0.05). CI: confidence interval; ER: estrogen receptor; PR: progesterone receptor; Her2: human epithelial growth factor receptor 2.

### Evaluate the predictive performance of the miRNA-based prognostic model

**Figure 3:**
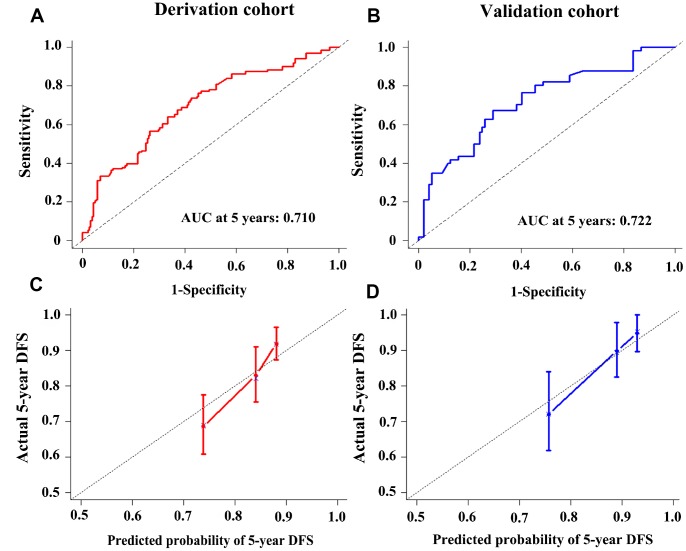
**Time-dependent receiver operating characteristic curves at 5-years based on the miRNA-based prognostic model in the derivation cohort** (**A**) and validation cohort (**B**). Calibration curves of the miRNA-based prognostic model in the derivation cohort (**C**) and validation cohort (**D**).

**Figure 4:**
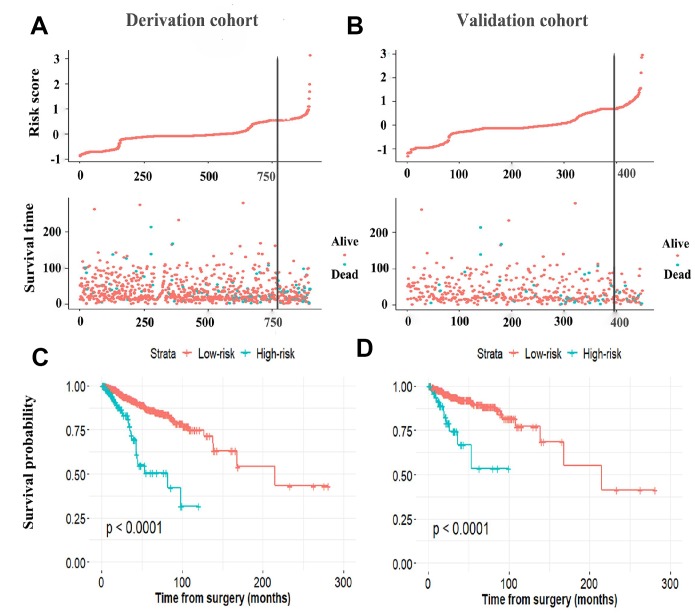
**The distribution of risk score, DFS, and DFS status in the derivation cohort** (**A**) and validation cohort (**B**). The black line indicates the optimal cutoff point of the nomogram score used to stratify patients into the low- and high-risk group. Kaplan–Meier curves of the low- and high-risk patients based on the miRNA-based prognostic model in the derivation cohort (**C**) and validation cohort (**D**). DFS, disease-free survival.

**Figure 5:**
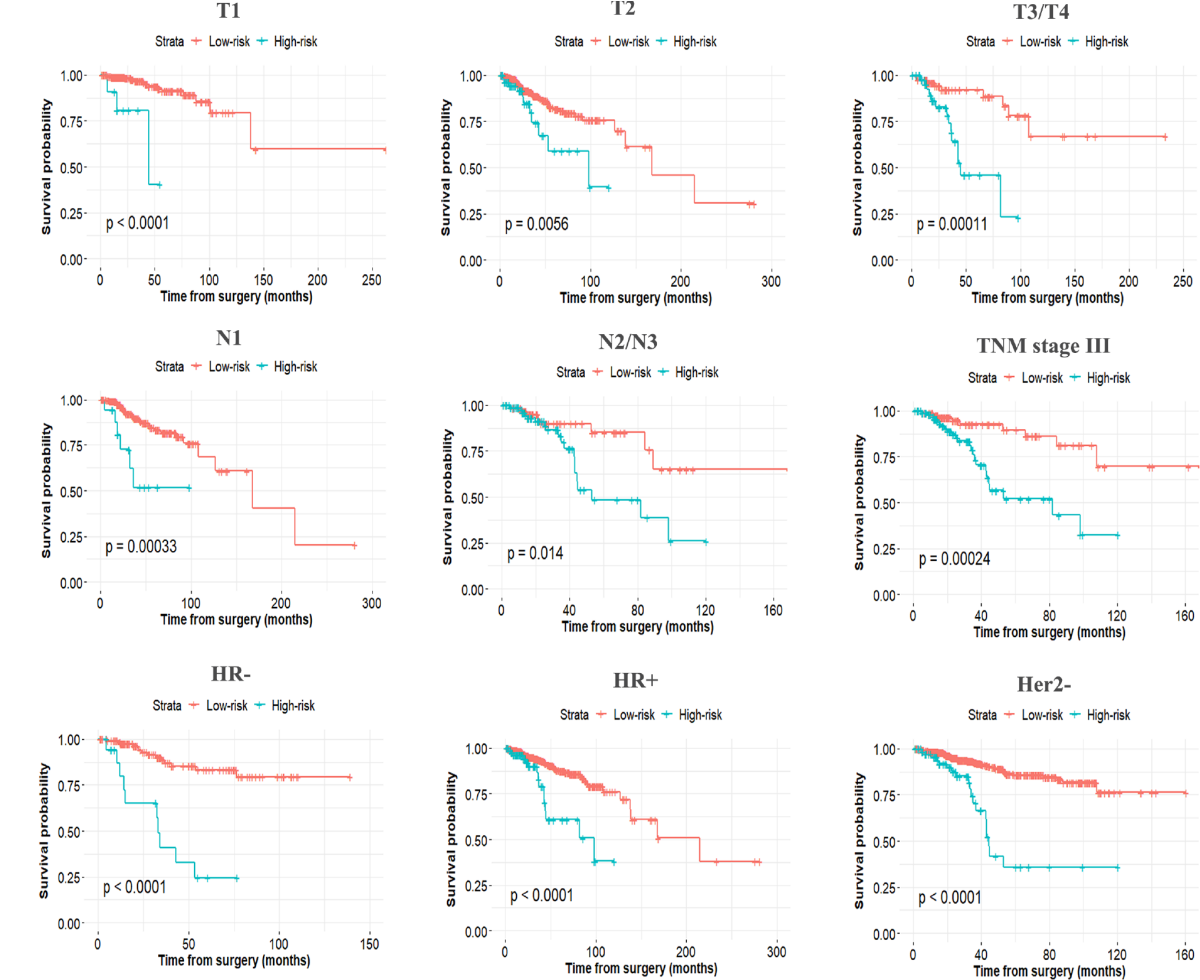
**Stratified analysis of the miRNA-based prognostic model for breast cancer patients in T stage, N stage, TNM stage, HR, and Her2 status.**

**Figure 6:**
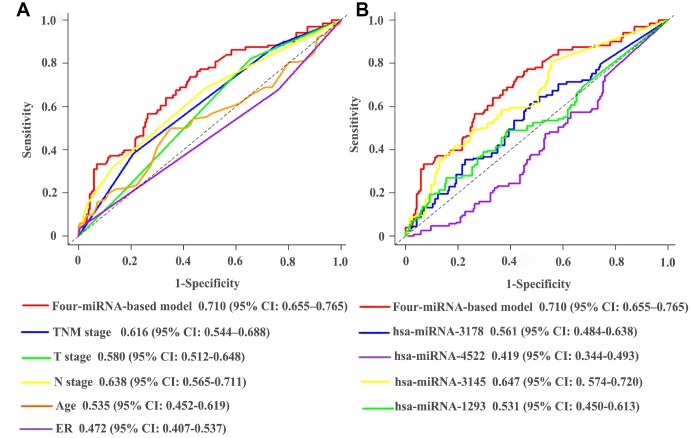
**Comparisons of the predictive accuracy at 5-years DFS using time-dependent receiver operating characteristic curves in miRNA-based model with clinical risk factors** (**A**), and miRNA-based model with single prognostic miRNA (**B**). DFS, disease-free survival.

The established miRNA-based prognostic nomogram was shown in Figure 2. The area under the curve (AUC) values of the miRNA-based model at 5 years were 0.710 (95% CI: 0.655–0.765) and 0.722 (95% CI: 0.604–0.841) in the derivation and internal validation cohort, which indicated good predictive accuracy of this nomogram (Figure 3A–3B). Moreover, the calibration plots of the miRNA-based nomogram fitted well in the two independent cohorts, which demonstrated good calibration ability of the model (Figure 3C–3D). Based on the nomogram scoring system, each patient acquired a total nomogram score. With the optimal cutoff total scores (1.7255) determined via X-tile software [22], patients were stratified into the low-risk group (n=778) and high-risk group (n=119) in the derivation set. With the same cutoff scores, patients were divided into the low-risk group (n=397) and high-risk group (n=52) in the internal validation set. We also observed the distribution of risk scores, DFS, DFS statuses in the two independent data sets (Figure 4A–4B). In addition, on the basis of risk stratification system, Kaplan–Meier curves were performed in both the derivation and the internal validation cohort, which demonstrated that patients in the high-risk group had poorer DFS than those in the low-risk group (*P* < 0.0001, Figure 4C–4D). The 5-year DFS rates of 897 patients were 0.862 (95% CI: 0.820–0.895) and 0.506 (95% CI: 0.339–0.652) in the low- and high-risk groups, respectively (*P* < 0.0001). Besides, we conducted effective risk stratification analyses in BC patients with T stage, N stage, TNM stage, hormone receptor (HR) and human epithelial growth factor receptor 2 (Her2) status. And patients in the low-risk group had significantly better DFS than those in the high-risk group in T1 (*P* < 0.0001), T2 (*P* = 0.0056), T3/T4 (*P* = 0.00011), TNM stage III (*P* = 0.00024), N1 (*P* = 0.00033), N2/N3 (*P* = 0.014), HR– (*P* < 0.0001), HR+ (*P* < 0.0001), Her2– (*P* < 0.0001) and Her2+ (*P* = 0.032) (Figure 5). Additionally, time-dependent receiver operating characteristic (ROC) analyses indicated that the miRNA-based prognostic model had better predictive performance than any clinical risk factors, or single prognostic miRNA alone in Figure 6. In terms of predictive accuracy, the miRNA-based prognostic nomogram was distinctly greater than that of the TNM stage (AUC: 0.710 vs 0.667, *P* < 0.0001).

### Functional enrichment analysis of predicted target genes

**Figure 7:**
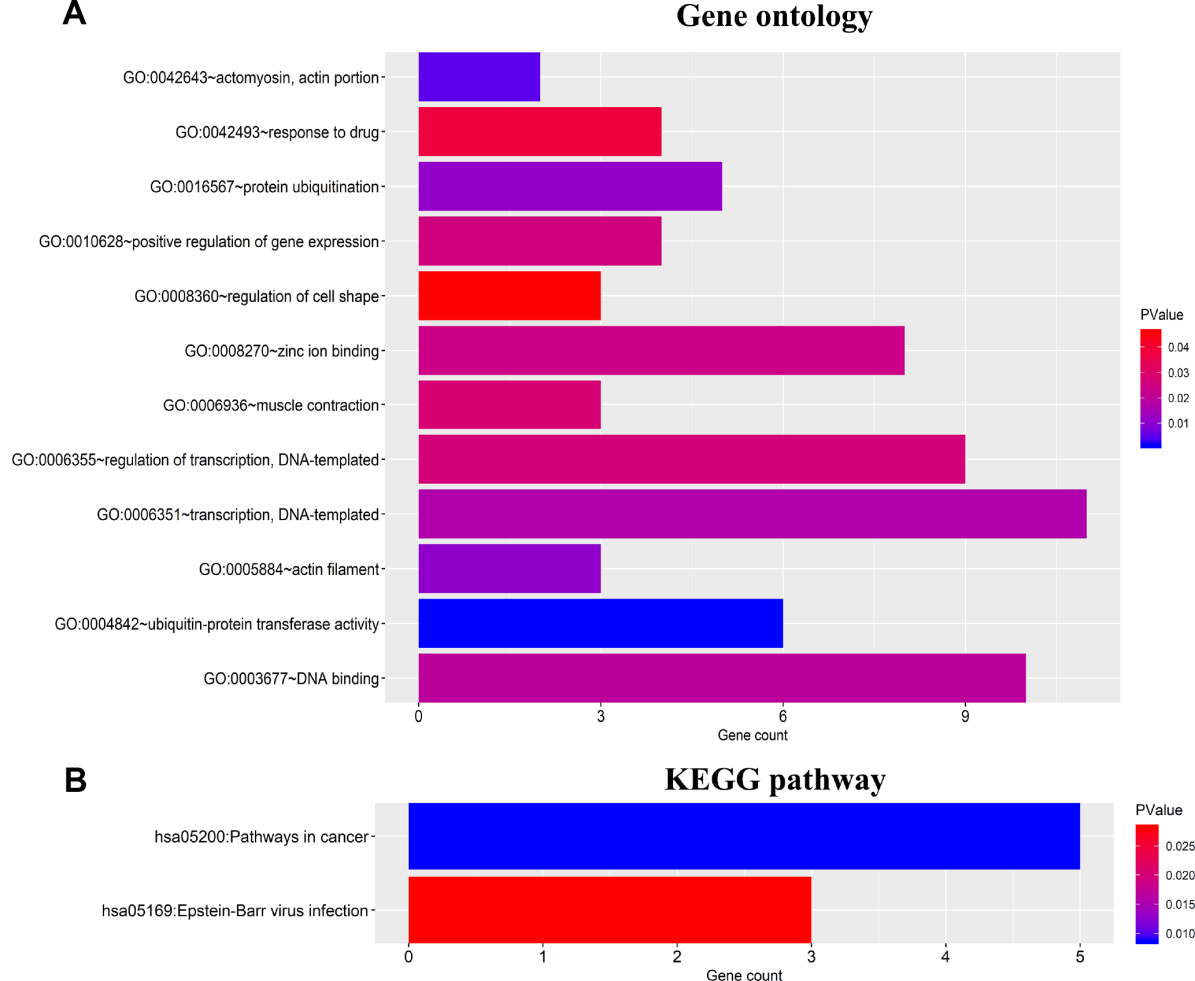
**Functional enrichment analysis for 46 target genes of the four miRNAs.** (**A**) Gene ontology (GO) enrichment analysis. (**B**) Kyoto Encyclopedia of Genes and Genomes analyses (KEGG) enrichment analysis. The x-axis indicates the number of genes, and the y-axis represents the GO terms and KEGG pathway names. The color represents the *P* value.

To further identify the potential cellular biological functions and mechanisms of the four prognostic miRNAs, 46 target genes were predicted using three databases, including TargetScan, miRTarBase and miRDB. Gene ontology (GO) analysis revealed that these genes were related with protein binding, cytoplasm and nucleus (Figure 7A). Kyoto Encyclopedia of Genes and Genomes analysis (KEGG) pathways analyses found that these genes mainly enrichment in cancer-related pathways and Epstein-Barr virus infection (Figure 7B).

## DISCUSSION

Prognostic evaluation is vital for making appropriate treatment decisions. Because the traditional TNM stage is mainly based on anatomical information, it is unable to achieve adequate assessment of disease recurrence in BC patients. Therefore, in the current study, we built and validated a novel model integrating miRNA recurrence-related signature and TNM stage to improve individualized risk prediction of DFS in BC patients. This tool conducted well as supported by the good predictive accuracy (AUC > 0.7) in the derivation and internal validation sets, respectively. Moreover, the calibration curves illustrated the good agreements between nomogram prediction and actual observations. Combination of miRNA recurrence-related signature and TNM stage was superior to TNM stage, indicating that the miRNA recurrence-related signature added the prognostic value of TNM stage. Furthermore, this miRNA-based nomogram could significantly stratify patients into the low- and high-risk group independent of the same TNM stage. Such accurate risk stratification could allow oncologists to identify the high-risk patients for aggressive therapy to improve BC prognosis.

Previous researches about miRNAs demonstrated that the miRNA-based signature is a crucial predictor for tumor recurrence [14, 16, 19, 20, 23–30]. Gong et al constructed a 10-miRNA-based classifier to predict recurrence in hormone receptor (HR)+ Her2- BC patients [19]. However, this study did not combine 10-miRNA signature with TNM stage to build a prognostic nomogram and was limited by small miRNA expression profiling. Prognostic nomogram, which comprise the visualization of statistical models, has potential to achieve a more individualized risk prediction of survival outcomes on the basis of combination of different prognostic variables [7, 21]. A large dataset of TCGA project provides us with a comprehensive foundation to mine multi-miRNA-based prognostic signature. Thus, a novel prognostic nomogram based on TCGA database, which incorporates multi-miRNA-based and TNM stage, is essential to make individualized estimates of 5-year DFS in BC patients. On the other hand, some previous studies have been limited by small number of miRNAs screened, small sample sizes and lack of independent validation [31–33]. It should be pointed out that the sample size influences the result of statistical significance [21]. As a result, these previous studies may not have an adequate sample size to identify a significant effect estimate. Thus, our study is more reliable and relevant from those published in several previous studies [31–33].

There are some limitations in the study. Firstly, we lack of experimental study to explain the biological implications of the miRNA recurrence-related signature. Thus, the molecular mechanism of these miRNAs should be investigated in further study. Secondly, the miRNA-based prognostic nomogram needs to be further validated by a prospective, large-scale multicenter study before it can be applied in clinical practice. Thirdly, the TCGA database lacks of postoperative information (adjuvant chemotherapy, radiotherapy). Hence, we could not identify the low-risk patients to tailor adjuvant therapy and foresee which patients are likely to benefit from adjuvant chemotherapy.

## CONCLUSIONS

In summary, a novel prognostic nomogram incorporating miRNA recurrence-related signature and TNM stage was established and internally validated to improve individualized risk estimation of 5-year DFS and make accurate risk stratification in BC patients. This easy-to-use tool can help clinicians to predict 5-year DFS and easily select proper patients who are in need of a specific therapeutic strategy.

## MATERIALS AND METHODS

### Participants and study design

Data for selected samples of 1044 BC samples and 102 adjacent normal tissues were downloaded from TCGA database. Inclusion criteria were as follows: (i) histological diagnosis of invasive BC; (ii) complete follow-up data and miRNA expression profile available. Then, a total of 897 patients were included in this study. According to a computer-generated allocation numbers, 449 patients as validation cohort were randomly selected from the 897 patients (derivation cohort). Because the data were all publicly derived from the TCGA project, approval by our institutional ethics committees was not needed.

### Establishment of multi-miRNA-based risk score and prognostic nomogram

To screen out the DEMs between 1044 BC samples and 102 adjacent normal tissues, we defined DEMs with a FDR < 0.05 and |log2FC)| ≥1. Then, univariate CPHR analysis was conducted to found the association between each DEMs, clinical risk factors and DFS (*P* < 0.05). Multivariate CPHR analysis was used to confirm the independent variables (*P* < 0.05). Thus, independent prognostic DEMs were selected to build a multi-miRNA-based risk score. And the multi-miRNA-based risk score = sum of coefficients × expression level of miRNAs. Furthermore, to make full use of the prognostic miRNAs, a novel model integrating the multi-miRNA-based signature and clinical factors to improve survival prediction in BC patients.

### Assessment of the multi-miRNA-based prognostic nomogram

To further evaluate the risk stratification ability of the multi-miRNA-based nomogram, we classified patients into the high- and low-risk subgroups according to the optimal cutoff nomogram score determined by X-tile plot [22]. Moreover, the predictive accuracy of the multi-miRNA-based model was calculated via AUC based on time-dependent ROC analysis [34]. Finally, calibration plot was performed to assess the calibration ability of the multi-miRNA-based nomogram.

### Target gene prediction and functional enrichment analysis

We applied TargetScan, miRTarBase and miRDB to confirm the target genes of prognostic miRNAs [35–37]. Then, GO and KEGG pathway enrichment analyses were executed to analyze these target genes using the database for Annotation, Visualization, and Integrated Discovery 6.8 Bioinformatics Tool (DAVID 6.8) [38].

### Statistical analysis

The χ2 test and the Mann-Whitney U test were used to compare the differences of variables between the two data sets, when appropriate. Survival curves were conducted via the Kaplan-Meier method and compared via the log-rank test. A threshold *P* < 0.05 was determined as statistical significance. The optimal cut-off values of prognostic nomogram scores were confirmed using X-tile software, version 3.6.1 (Yale University, New Haven, CT, USA) [22]. Stata/ MP, version 14.0 (StataCorp LP, College Station, TX) and R version 3.4.4 were applied to the statistical analyses.

## References

[1] Bray F, Ferlay J, Soerjomataram I, Siegel RL, Torre LA, Jemal A. Global cancer statistics 2018: GLOBOCAN estimates of incidence and mortality worldwide for 36 cancers in 185 countries. CA Cancer J Clin. 2018; 68:394–424. 10.3322/caac.21492 30207593

[2] Chen X, Zhang G, Chen B, Wang Y, Guo L, Cao L, Ren C, Wen L, Liao N. Association between histone lysine methyltransferase KMT2C mutation and clinicopathological factors in breast cancer. Biomed Pharmacother. 2019; 116:108997. 10.1016/j.biopha.2019.108997 31146111

[3] Chen B, Tang H, Chen X, Zhang G, Wang Y, Xie X, Liao N. Transcriptomic analyses identify key differentially expressed genes and clinical outcomes between triple-negative and non-triple-negative breast cancer. Cancer Manag Res. 2018; 11:179–90. 10.2147/CMAR.S187151 30613165 PMC6306052

[4] Zhang G, Wang Y, Chen B, Guo L, Cao L, Ren C, Wen L, Li K, Jia M, Li C, Mok H, Chen X, Wei G, et al. Characterization of frequently mutated cancer genes in Chinese breast tumors: a comparison of Chinese and TCGA cohorts. Ann Transl Med. 2019; 7:179–179. 10.21037/atm.2019.04.23 31168460 PMC6526269

[5] Berry DA, Cronin KA, Plevritis SK, Fryback DG, Clarke L, Zelen M, Mandelblatt JS, Yakovlev AY, Habbema JD, Feuer EJ, and Cancer Intervention and Surveillance Modeling Network (CISNET) Collaborators. Effect of screening and adjuvant therapy on mortality from breast cancer. N Engl J Med. 2005; 353:1784–92. 10.1056/NEJMoa050518 16251534

[6] Ni YB, Tsang JY, Chan SK, Tse GM. A novel morphologic-molecular recurrence predictive model refines traditional prognostic tools for invasive breast carcinoma. Ann Surg Oncol. 2014; 21:2928–33. 10.1245/s10434-014-3691-9 24743910

[7] Balachandran VP, Gonen M, Smith JJ, DeMatteo RP. Nomograms in oncology: more than meets the eye. Lancet Oncol. 2015; 16:e173–80. 10.1016/S1470-2045(14)71116-7 25846097 PMC4465353

[8] Bartel DP. MicroRNAs: genomics, biogenesis, mechanism, and function. Cell. 2004; 116:281–97. 10.1016/S0092-8674(04)00045-5 14744438

[9] Esquela-Kerscher A, Slack FJ. Oncomirs - microRNAs with a role in cancer. Nat Rev Cancer. 2006; 6:259–69. 10.1038/nrc1840 16557279

[10] Kawaguchi T, Yan L, Qi Q, Peng X, Edge SB, Young J, Yao S, Liu S, Otsuji E, Takabe K. Novel MicroRNA-Based Risk Score Identified by Integrated Analyses to Predict Metastasis and Poor Prognosis in Breast Cancer. Ann Surg Oncol. 2018; 25:4037–46. 10.1245/s10434-018-6859-x 30311168 PMC6245576

[11] Zhu R, Lin W, Zhao W, Fan F, Tang L, Hu YA. 4-microRNA signature for survival prognosis in pediatric and adolescent acute myeloid leukemia. J Cell Biochem. 2019; 120:3958–3968. 10.1002/jcb.27679 30242879

[12] Zhao Y, Schetter AJ, Yang GB, Nguyen G, Mathé EA, Li P, Cai H, Yu L, Liu F, Hang D, Yang H, Wang XW, Ke Y, Harris CC. microRNA and inflammatory gene expression as prognostic marker for overall survival in esophageal squamous cell carcinoma. Int J Cancer. 2013; 132:2901–09. 10.1002/ijc.27954 23175214 PMC6503976

[13] Zhang JX, Song W, Chen ZH, Wei JH, Liao YJ, Lei J, Hu M, Chen GZ, Liao B, Lu J, Zhao HW, Chen W, He YL, et al. Prognostic and predictive value of a microRNA signature in stage II colon cancer: a microRNA expression analysis. Lancet Oncol. 2013; 14:1295–306. 10.1016/S1470-2045(13)70491-1 24239208

[14] Yang Y, Qu A, Zhao R, Hua M, Zhang X, Dong Z, Zheng G, Pan H, Wang H, Yang X, Zhang Y. Genome-wide identification of a novel miRNA-based signature to predict recurrence in patients with gastric cancer. Mol Oncol. 2018; 12:2072–84. 10.1002/1878-0261.12385 30242969 PMC6275280

[15] Tang XR, Li YQ, Liang SB, Jiang W, Liu F, Ge WX, Tang LL, Mao YP, He QM, Yang XJ, Zhang Y, Wen X, Zhang J, et al. Development and validation of a gene expression-based signature to predict distant metastasis in locoregionally advanced nasopharyngeal carcinoma: a retrospective, multicentre, cohort study. Lancet Oncol. 2018; 19:382–93. 10.1016/S1470-2045(18)30080-9 29428165

[16] Shu X, Hildebrandt MA, Gu J, Tannir NM, Matin SF, Karam JA, Wood CG, Wu X. MicroRNA profiling in clear cell renal cell carcinoma tissues potentially links tumorigenesis and recurrence with obesity. Br J Cancer. 2017; 116:77–84. 10.1038/bjc.2016.392 27907930 PMC5220154

[17] Sana J, Radova L, Lakomy R, Kren L, Fadrus P, Smrcka M, Besse A, Nekvindova J, Hermanova M, Jancalek R, Svoboda M, Hajduch M, Slampa P, et al. Risk Score based on microRNA expression signature is independent prognostic classifier of glioblastoma patients. Carcinogenesis. 2014; 35:2756–62. 10.1093/carcin/bgu212 25322872

[18] Li X, Shi Y, Yin Z, Xue X, Zhou B. An eight-miRNA signature as a potential biomarker for predicting survival in lung adenocarcinoma. J Transl Med. 2014; 12:159. 10.1186/1479-5876-12-159 24893932 PMC4062505

[19] Gong C, Tan W, Chen K, You N, Zhu S, Liang G, Xie X, Li Q, Zeng Y, Ouyang N, Li Z, Zeng M, Zhuang S, et al. Prognostic Value of a BCSC-associated MicroRNA Signature in Hormone Receptor-Positive HER2-Negative Breast Cancer. EBioMedicine. 2016; 11:199–209. 10.1016/j.ebiom.2016.08.016 27566954 PMC5049991

[20] Bagnoli M, Canevari S, Califano D, Losito S, Maio MD, Raspagliesi F, Carcangiu ML, Toffoli G, Cecchin E, Sorio R, Canzonieri V, Russo D, Scognamiglio G, et al, and Multicentre Italian Trials in Ovarian cancer (MITO) translational group. Development and validation of a microRNA-based signature (MiROvaR) to predict early relapse or progression of epithelial ovarian cancer: a cohort study. Lancet Oncol. 2016; 17:1137–46. 10.1016/S1470-2045(16)30108-5 27402147

[21] Iasonos A, Schrag D, Raj GV, Panageas KS. How to build and interpret a nomogram for cancer prognosis. J Clin Oncol. 2008; 26:1364–70. 10.1200/JCO.2007.12.9791 18323559

[22] Camp RL, Dolled-Filhart M, Rimm DL. X-tile: a new bio-informatics tool for biomarker assessment and outcome-based cut-point optimization. Clin Cancer Res. 2004; 10:7252–59. 10.1158/1078-0432.CCR-04-0713 15534099

[23] Liu N, Cui RX, Sun Y, Guo R, Mao YP, Tang LL, Jiang W, Liu X, Cheng YK, He QM, Cho WC, Liu LZ, Li L, Ma J. A four-miRNA signature identified from genome-wide serum miRNA profiling predicts survival in patients with nasopharyngeal carcinoma. Int J Cancer. 2014; 134:1359–68. 10.1002/ijc.28468 23999999

[24] Liu N, Chen NY, Cui RX, Li WF, Li Y, Wei RR, Zhang MY, Sun Y, Huang BJ, Chen M, He QM, Jiang N, Chen L, et al. Prognostic value of a microRNA signature in nasopharyngeal carcinoma: a microRNA expression analysis. Lancet Oncol. 2012; 13:633–41. 10.1016/S1470-2045(12)70102-X 22560814

[25] Ji D, Qiao M, Yao Y, Li M, Chen H, Dong Q, Jia J, Cui X, Li Z, Xia J, Gu J. Serum-based microRNA signature predicts relapse and therapeutic outcome of adjuvant chemotherapy in colorectal cancer patients. EBioMedicine. 2018; 35:189–97. 10.1016/j.ebiom.2018.08.042 30166271 PMC6156709

[26] Han ZB, Zhong L, Teng MJ, Fan JW, Tang HM, Wu JY, Chen HY, Wang ZW, Qiu GQ, Peng ZH. Identification of recurrence-related microRNAs in hepatocellular carcinoma following liver transplantation. Mol Oncol. 2012; 6:445–57. 10.1016/j.molonc.2012.04.001 22552153 PMC5528352

[27] Buffa FM, Camps C, Winchester L, Snell CE, Gee HE, Sheldon H, Taylor M, Harris AL, Ragoussis J. microRNA-associated progression pathways and potential therapeutic targets identified by integrated mRNA and microRNA expression profiling in breast cancer. Cancer Res. 2011; 71:5635–45. 10.1158/0008-5472.CAN-11-0489 21737487

[28] Bai F, Zhou H, Ma M, Guan C, Lyu J, Meng QH. A novel RNA sequencing-based miRNA signature predicts with recurrence and outcome of hepatocellular carcinoma. Mol Oncol. 2018; 12:1125–37. 10.1002/1878-0261.12315 29719937 PMC6026871

[29] Krishnan P, Ghosh S, Wang B, Li D, Narasimhan A, Berendt R, Graham K, Mackey JR, Kovalchuk O, Damaraju S. Next generation sequencing profiling identifies miR-574-3p and miR-660-5p as potential novel prognostic markers for breast cancer. BMC Genomics. 2015; 16:735. 10.1186/s12864-015-1899-0 26416693 PMC4587870

[30] Jacob H, Stanisavljevic L, Storli KE, Hestetun KE, Dahl O, Myklebust MP. A four-microRNA classifier as a novel prognostic marker for tumor recurrence in stage II colon cancer. Sci Rep. 2018; 8:6157. 10.1038/s41598-018-24519-4 29670141 PMC5906690

[31] Papadaki C, Stratigos M, Markakis G, Spiliotaki M, Mastrostamatis G, Nikolaou C, Mavroudis D, Agelaki S. Circulating microRNAs in the early prediction of disease recurrence in primary breast cancer. Breast Cancer Res. 2018; 20:72. 10.1186/s13058-018-1001-3 29996899 PMC6042266

[32] Huo D, Clayton WM, Yoshimatsu TF, Chen J, Olopade OI. Identification of a circulating microRNA signature to distinguish recurrence in breast cancer patients. Oncotarget. 2016; 7:55231–48. 10.18632/oncotarget.10485 27409424 PMC5342414

[33] Pérez-Rivas LG, Jerez JM, Carmona R, de Luque V, Vicioso L, Claros MG, Viguera E, Pajares B, Sánchez A, Ribelles N, Alba E, Lozano J. A microRNA signature associated with early recurrence in breast cancer. PLoS One. 2014; 9:e91884. 10.1371/journal.pone.0091884 24632820 PMC3954835

[34] Heagerty PJ, Lumley T, Pepe MS. Time-dependent ROC curves for censored survival data and a diagnostic marker. Biometrics. 2000; 56:337–44. 10.1111/j.0006-341X.2000.00337.x 10877287

[35] Agarwal V, Bell GW, Nam JW, Bartel DP. Predicting effective microRNA target sites in mammalian mRNAs. eLife. 2015; 4:4. 10.7554/eLife.05005 26267216 PMC4532895

[36] Wong N, Wang X. miRDB: an online resource for microRNA target prediction and functional annotations. Nucleic Acids Res. 2015; 43:D146–52. 10.1093/nar/gku1104 25378301 PMC4383922

[37] Chou CH, Shrestha S, Yang CD, Chang NW, Lin YL, Liao KW, Huang WC, Sun TH, Tu SJ, Lee WH, Chiew MY, Tai CS, Wei TY, et al. miRTarBase update 2018: a resource for experimentally validated microRNA-target interactions. Nucleic Acids Res. 2018; 46:D296–302. 10.1093/nar/gkx1067 29126174 PMC5753222

[38] Huang W, Sherman BT, Lempicki RA. Bioinformatics enrichment tools: paths toward the comprehensive functional analysis of large gene lists. Nucleic Acids Res. 2009; 37:1–13. 10.1093/nar/gkn923 19033363 PMC2615629

